# Commentary: The Affective Core of the Self: A Neuro-Archetypical Perspective on the Foundations of Human (and Animal) Subjectivity

**DOI:** 10.3389/fpsyg.2017.02098

**Published:** 2017-11-30

**Authors:** Gregory Bonn

**Affiliations:** General Studies, Psychology, King Fahd University of Petroleum and Minerals, Dhahran, Saudi Arabia

**Keywords:** consciousness, emotions, animal consciousness, self-awareness, affective neuroscience

This commentary argues that Alcaro, Carta, and Panksepp's model of the self, consciousness, and emotion is scientifically sound as well as ontologically preferable to other recently advocated theories (Alcaro et al., 2017). Alcaro et al.'s model locates the roots of self-processes within the same subcortical midline structures that Panksepp (1998) and Panksepp and Northoff (2009) identify as the bases for “primary-process” emotions. They argue that this emotional core provides the fundamental sense of subjectivity, or being-in-relation-to-something, upon which complex, self-referential experiences are built. Many neuroscientists and philosophers, however, contend that human consciousness and emotion should be conceived as clearly distinct from that of animals. A recent article by LeDoux and Brown (2017), for example, argues for a redefinition of the term emotion that requires human-like self-reflection: Because human experience involves the ability to reflect upon and label emotional self-states, they argue that experiences lacking these qualities cannot be called emotions.

It is agreed that human emotional experience must differ from that of other animals in many ways: Human qualitative experience involves layers of complexity and recursivity that are likely unique to our species. Narrowly defining consciousness and emotion to that which can be recalled and described, as Ledoux and to a lesser extent, Brown (2015) suggest is, in my view, ethically troubling. Alternatively, Alcaro et al. suggest that consciousness and emotion are multi-layered phenomena which have their roots in core affective experience. As such, many aspects of consciousness and emotional states are almost certainly shared between humans and other animals, while other, more self-reflective qualia probably are not. Granted, some aspects of human emotion must be unique, at the same time, however, much of human emotional experience is indescribable. Does this mean it doesn't qualify as emotion?

Ledoux and Brown define consciousness as “subjective experience, as opposed to the condition of simply being awake and responsive to sensory stimulation” (p. E2017) where “subjective experience” is operationalized as an adult human's ability to verbally report an experience. Outside the lab, this definition becomes problematic (Bonn, 2013). Does this mean that pre-verbal children or dementia patients who cannot accurately recall their state of mind from a few minutes ago are not conscious? Brown (2017) argues that, although he believes animals are conscious, if we believe in a robust cognitive unconscious we can't infer consciousness simply from behavior:Verbal report, thus, is necessary for us to be *sure* that consciousness is involved. The problem is, however, we also can't be sure that consciousness is *not* involved when verbal report is absent. Most people have had experiences that they would consider conscious which cannot be accurately described or recalled. Similarly, logical arguments aside, almost anyone who has observed canine or primate social behavior for any length of time would have difficulty insisting that there is not some level of consciousness involved; the same for pre-verbal children or dementia sufferers. The experience of healthy adult humans certainly differs qualitatively from other species as well as less high-functioning humans, but equating our definitions of consciousness and emotion with these differences soon becomes ethically untenable.

Much of this relates to Block's (2014) distinction between phenomenal consciousness and access consciousness. Essentially, there is a difference between having an experience and the abstract cognitive processing of that experience. Cognitive processing requires not just experience, but the ability to monitor that experience and label it for use in future operations; a consciousness of conscious experience. Most experiments operationalize consciousness as the abstract representation and report of experience. This alone is not necessarily a problem. A problem, however, lies in the assertion that conscious experience itself must be explicitly self-referential (e.g., Rochat, 2003; Baars, 2005). It can just as easily be argued that recollections are quite separate from experience (e.g., Kahneman and Riis, 2005) and even that self-referential processes often interfere with optimal experience (Csikszentmihalyi, 1990).

In my view, LeDoux and Brown (2017) go too far in contending that emotions require higher-order cognitive processing and, thus, that they are likely the exclusive domain of humans. In their model, for example, animals don't experience “fear”: They have a “defensive survival circuit” (p. E2107). Regardless of the type and form of physiological arousal involved, they argue that an affective experience cannot be called an emotion if it lacks an abstract self-representation. This view stands in contrast to decades of findings by Panksepp which show fundamental similarities in the structure and function of emotional circuits among all mammals (Watt, 2005). Primary-process emotions are, essentially, variously nuanced approach and avoidance impulses that occur in relationship to objects in the environment. This relatedness-to-objects, in Alcaro et al.'s view, requires some form of implicit sense of self. Such primal experiences of self and emotion would be much different from the cognitively elaborated forms experienced by humans. However, the tone and valence of emotional experiences in their view are established before human cognitive networks perform their appraisals and offer their feedback.

Humans, of course, incorporate many layers of complexity with, and modulate the intensity of, emotion through their networks of cognition. As Ledoux rightfully points out, human emotional dysfunction often results from problems with such cognitive processes: Treatment programs for humans, thus, demand close attention to the nature and workings of cognition. We can, however, acknowledge the many unique qualities of human emotion and experience without discounting the internal experiences of other species. At the same time, if we focus less narrowly on the cognitive aspects of emotion and consciousness perhaps we can better appreciate the depths of our own experiences. To paraphrase Kahneman and Riis (2005), there is a difference between living life and thinking about it. Redefining psychologically loaded terms such as emotion and consciousness so that they match with simple experimental protocols discounts the richness of experience. Alcaro et al., on the other hand, provide a scientifically defensible alternative which remains true to the intuitive, experiential and ethical conceptions of emotion and consciousness held by many.

## Author contributions

The author confirms being the sole contributor of this work and approved it for publication.

### Conflict of interest statement

The author declares that the research was conducted in the absence of any commercial or financial relationships that could be construed as a potential conflict of interest.
